# Prediction of severity and subtype of fibrosing disease using model informed by inflammation and extracellular matrix gene index

**DOI:** 10.1371/journal.pone.0240986

**Published:** 2020-10-23

**Authors:** Amin M. Cheikhi, Zariel I. Johnson, Dana R. Julian, Sarah Wheeler, Carol Feghali-Bostwick, Yvette P. Conley, James Lyons-Weiler, Cecelia C. Yates

**Affiliations:** 1 McGowan Institute for Regenerative Medicine, Pittsburgh, PA, United States of America; 2 Department of Health Promotion and Development, University of Pittsburgh School of Nursing, Pittsburgh, PA, United States of America; 3 Department of Pathology, University of Pittsburgh School of Medicine, Pittsburgh, PA, United States of America; 4 Department of Rheumatology & Immunology, Medical University of South Carolina, Charleston, SC, United States of America; 5 Genomic and Proteomic Core Laboratories, University of Pittsburgh, Pittsburgh, PA, United States of America; University of Texas McGowan Medical School at Houston, UNITED STATES

## Abstract

Fibrosis is a chronic disease with heterogeneous clinical presentation, rate of progression, and occurrence of comorbidities. Systemic sclerosis (scleroderma, SSc) is a rare rheumatic autoimmune disease that encompasses several aspects of fibrosis, including highly variable fibrotic manifestation and rate of progression. The development of effective treatments is limited by these variabilities. The fibrotic response is characterized by both chronic inflammation and extracellular remodeling. Therefore, there is a need for improved understanding of which inflammation-related genes contribute to the ongoing turnover of extracellular matrix that accompanies disease. We have developed a multi-tiered method using Naïve Bayes modeling that is capable of predicting level of disease and clinical assessment of patients based on expression of a curated 60-gene panel that profiles inflammation and extracellular matrix production in the fibrotic disease state. Our novel modeling design, incorporating global and parametric-based methods, was highly accurate in distinguishing between severity groups, highlighting the importance of these genes in disease. We refined this gene set to a 12-gene index that can accurately identify SSc patient disease state subsets and informs knowledge of the central regulatory pathways in disease progression.

## Introduction

Fibrosis results from continuous connective tissue remodeling during a reparative or reactive process, leading to disrupted tissue function in affected organs. The high mortality rate from fibrosing diseases is a multifaceted health issue in the developed world [1] that continues to demand further exploitation. Progress in this area requires reverse translation of clinical findings that inform preclinical studies, and re-validation and/or generation of existing or new animal models.

Fundamental to the challenges in generating effective treatments for the majority of patients is the heterogeneity of fibrosing diseases’ symptom patterns, progression, and severity. Current research has focused on the causes of fibrosis, the discovery of fibrosis-associated biomarkers, and the associations between fibrosis and disease [2–5]. Further inquiry is needed to gain a deeper understanding of progression of the fibrosing state. Notably, addressing the heterogeneity of fibrosing diseases is essential in providing a clear link between the multifaceted genomic and phenotypic changes of fibrosis.

Promisingly, new high-throughput 'omics' technologies are gaining traction as enablers of personalized medicine advance at a detailed molecular level, and as such could aid at combining data-driven inductive and symptom-based deductive approaches to accurately represent clinical fibrosis course. An exemplar of heterogeneous fibrosing diseases that can benefit from multivariate data analysis of high-dimensional multiset omics data, and the generation of valid and predictive models for insightful interpretation, is systemic sclerosis (scleroderma, SSc). SSc is a rare chronic disease, of still unknown cause, characterized by multi-organ diffuse fibrosis and vascular abnormalities.

During the SSc fibrotic process, a complex combination of cytokines, chemokines, growth factors, proteases, and extracellular matrix (ECM) constituents are secreted by dermal and resident epithelial cells, all of which add to the inflammatory infiltrate. The linkage of uncontrolled accumulation of ECM, a hallmark of fibrosis, with alterations in inflammatory mediators is concordant with a growing number of studies [6–8]. We and others have shown that a specific chemokines-driven multiscale signaling network (1) promotes attraction of inflammatory cells, (2) directs actions on various target cell types, (3) regulates angiogenesis, and (4) orchestrates tissue remodeling. This polyfunctional heterogeneity of secretions of chemokines and their receptors [9–12] is further evidenced by a number of studies linking the deregulation of chemokine receptor-specific levels to distinct organ and tissue fibrotic cues [11, 13–22].

Clinically, SSc is divided into two subtypes: a more progressive diffuse (dSSc) form and a limited (lSSc) form, depending on the extent of skin fibrosis. This heterogeneity has constrained current treatments that modestly benefit only a subset of patients and hindered predictive analytics of clinical outcomes [23].

The current “gold standard” for assessing severity of SSc in skin is a physical diagnostic test, the modified Rodnan skin score (mRSS). Biologically, the levels of chemokines and their receptors are often elevated in the serum of SSc patients, and fibroblasts (the master regulators of ECM production) from patients show altered chemokine signaling [24–27]. Thus, it is plausible that the variation in gene signatures coding for the extracellular matrix and inflammatory pathways is a reflection of the inherent biology of a given fibrosing disease, representing the pace of SSc instructive cues and hence clinical disease course as captured by mRSS skin score for diagnosis of disease severity.

Using several publicly available datasets, we have applied a novel method, the unsupervised efficiency analysis (UEA), to couple gene signatures to disease pathology and severity based on the stratification of patient-specific indicators of disease progression and outcome. The UEA compares differences in the percentage of overlapping of genes between two disease subsets. Datasets were first analyzed using caGEDA tool [28], which measures microarrays differential gene expression. Then we used the resulting differentially expressed genes to predict disease severity or clinical subtype using a Naïve Bayes classifier and to investigate their associated pathways. Further molecular stratification was used to develop score indices from genes known to be associated with SSc, chronic inflammation, fibrosis, and related canonical pathways. This study provides a principled framework for causal effects estimation from complex high-dimensional data using model informed by inflammation and extracellular matrix gene index related to organ and tissue-specific fibrotic cues. Using know key immuno-modulatory and extracellular matrix genes involved in the progression of SSc we have established a panel of 12-genes that could predict disease state with high accuracy to identify three‐way relationships between SSc phenotypes, genes and skin score.

## Results

The objectives of our present study are to test the correlation between chemokines and ECM genes in the samples from patient skin biopsies and identify the genes coding for chemokine genes that govern fibrosis-related alterations in key ECM genes in these patients. To meet this goal, we used a multi-tiered approach which included both unsupervised identification of differentially expressed genes, as well as a second phase that interrogated genes known to be involved in chemokine signaling and ECM production. In both phases, we compared between healthy and dSSc, and between dSSc and lSSc. We first identified a suitable dataset, consisting of gene profile information of skin biopsies using microarrays, which had been deposited to the publicly accessible National Center for Biotechnology Information GEO Database (Accession No. GSE9285) [29]. The sample population includes healthy patients as well as patients diagnosed with dSSc, lSSc, and morphea, with a wide range of mRSS varying across donor demographics (S1 Table). This dataset captures several elements of patient level heterogeneity, including patients with various combinations of gender, age, and racial background.

### Age and skin score association with disease type

**Fig 1:**
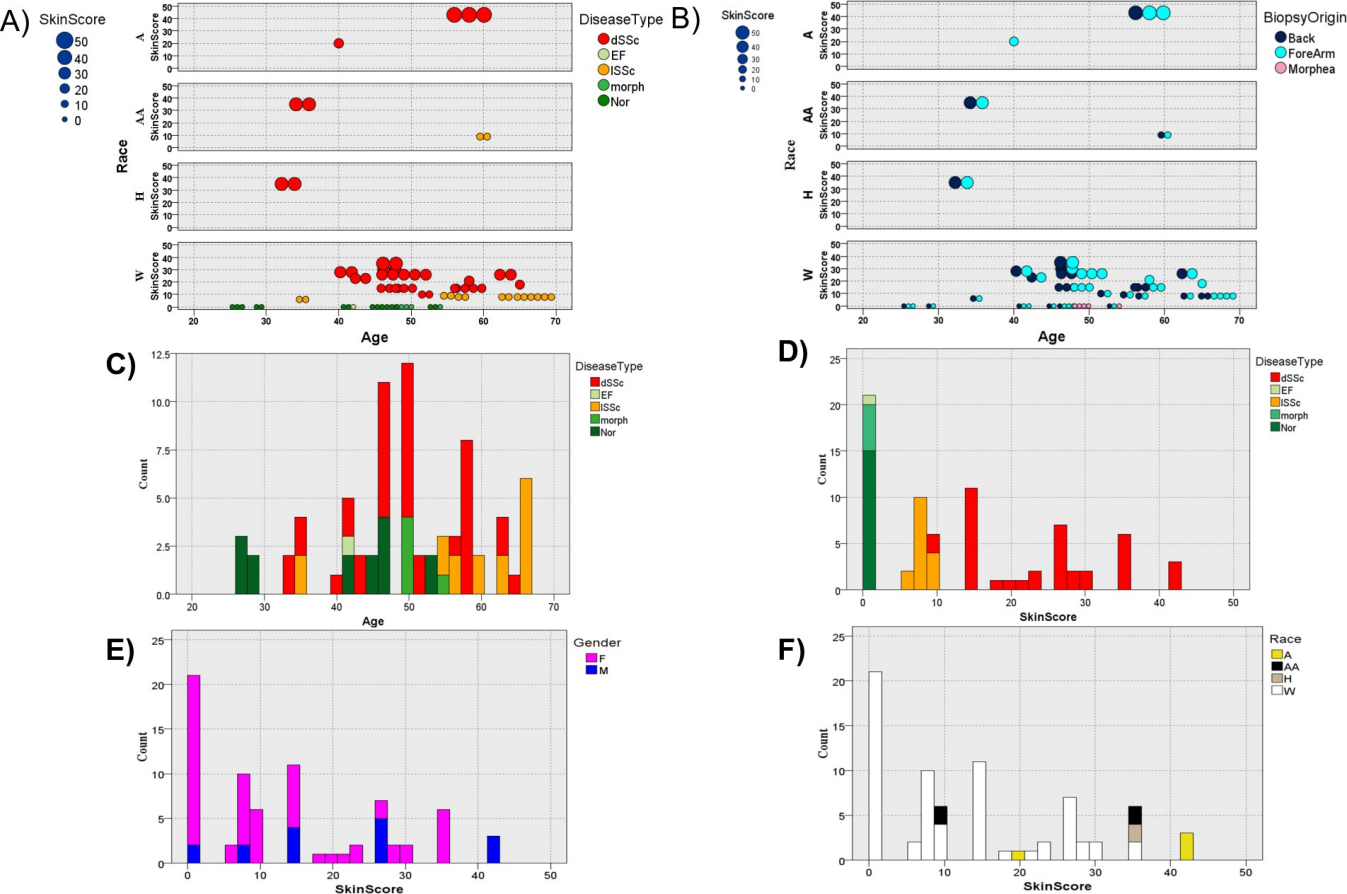
Qualitative and quantitative analysis of demographic and clinical characteristics of donor biopsies from microarray gene expression of patient skin biopsies. Shown are bubble charts reflecting the magnitude of the skin score given the race and age of the donors as function of the (A) disease type and (B) biopsy origin as well as the (C) distribution of donor age and disease type or (D) and skin scores as a function of the disease type, gender and race (A = Asian, AA = African American, H = Hispanic, W = White) respectively. Disease type, biopsy origin, race, and sex are color-coordinated, and the size of the bubbles indicate the magnitude of the skin score.

Qualitative and quantitative analysis of clinical features of patients from which skin biopsies for microarray gene expression analysis revealed an intricate interplay between the age of donors and the disease type, as reflected by (Fig 1A–1C) the preponderance of the dSSc type in the most geriatric donors and (Fig 1D, left panel) the typically high skin scores of the dSSc type. In contrast, the biopsy origin appears to have no bearing on the interplay of skin score and demographics (Fig 1B). However, in this study skin score dependence on gender and race, is less conclusive due to gender and race underrepresentation (Fig 1D, middle and right panels).

**Fig 2:**
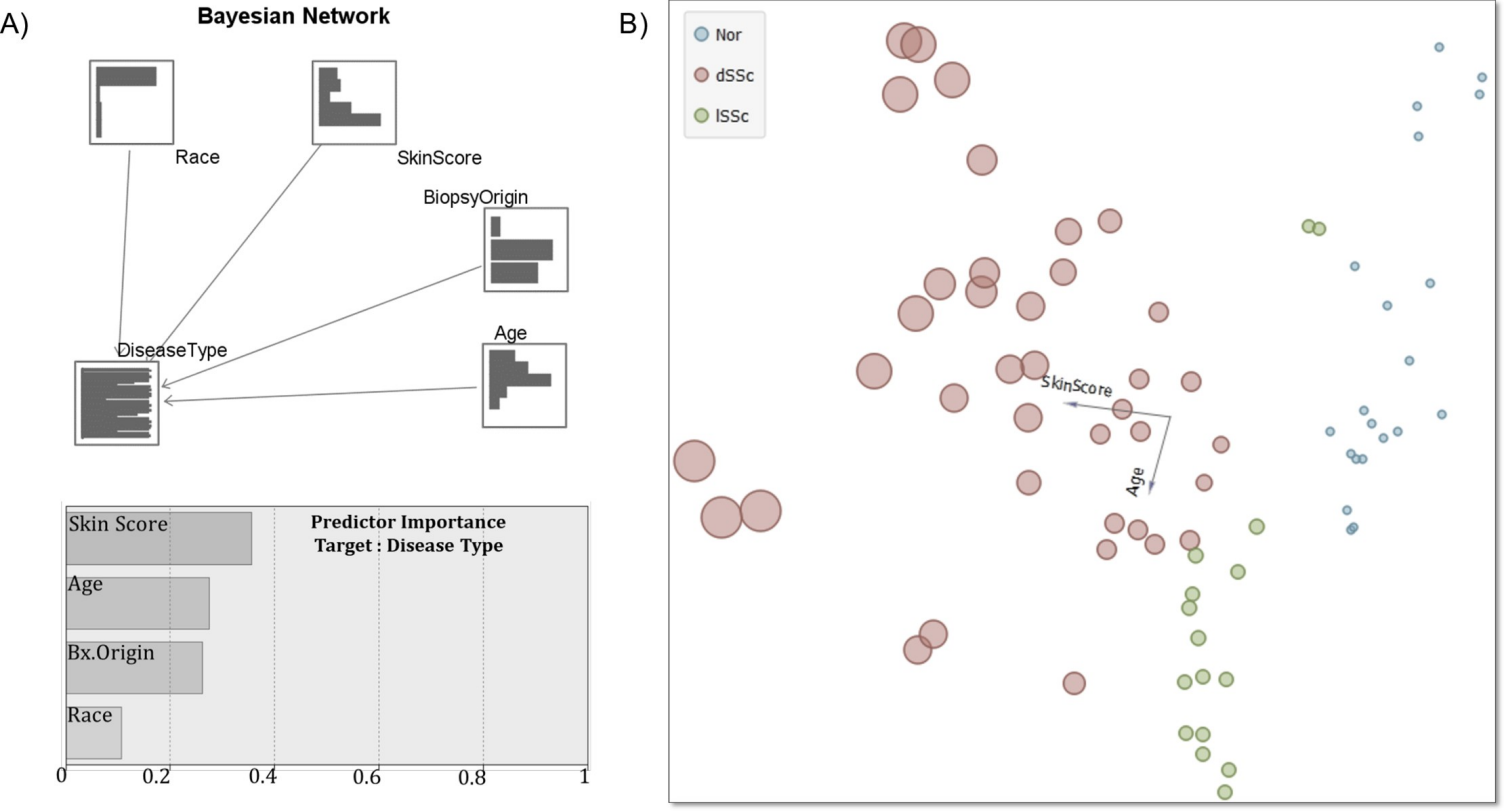
Conditional dependency between demographic and clinical characteristics of donor biopsies. (A) A simple Bayesian network model encoding the conditional probability between disease type classification as the target variable on other characteristics as predictors, and the relative predictor importance. The node focuses on Tree Augmented Naïve Bayes (TAN) and Markov Blanket networks that are primarily used for classification. (B) Linear projection methods using principal component analysis of disease type-labeled data showing the skin score/age two-dimensional projection where instances of different classes are best separated.

Bayesian Network was constructed to build a probability model by combining dataset features used in Milano et al. study features and to establish the likelihood of occurrences by using seemingly unlinked attributes. The model displays the interconnection of SSc disease subtype and other factors, such as skin score, age, race, and the origin of biopsy (Fig 2). Amongst those conditions, race demonstrated the lowest level of interdependency, while age and skin score stood as the highest predictors (Fig 2A and S2 Table) of SSc subtype.

The linear projection model developed by Koren et al. [30] which integrates data coordinates with pairwise similarities and/or differences to create a linear transformation displaying the separation and infrastructure between data clusters. Following Koren et al. methods, visual linear transformation of age dependency on SSc disease subtype, exposes definitive clustering of higher skin scores in older dSSc patients (Fig 2B).

### Genomic profiles of healthy vs. dSSc patients differ in their expression of matrix and growth factor signaling genes while dSSc vs. lSSc have a wide range of functions

**Fig 3:**
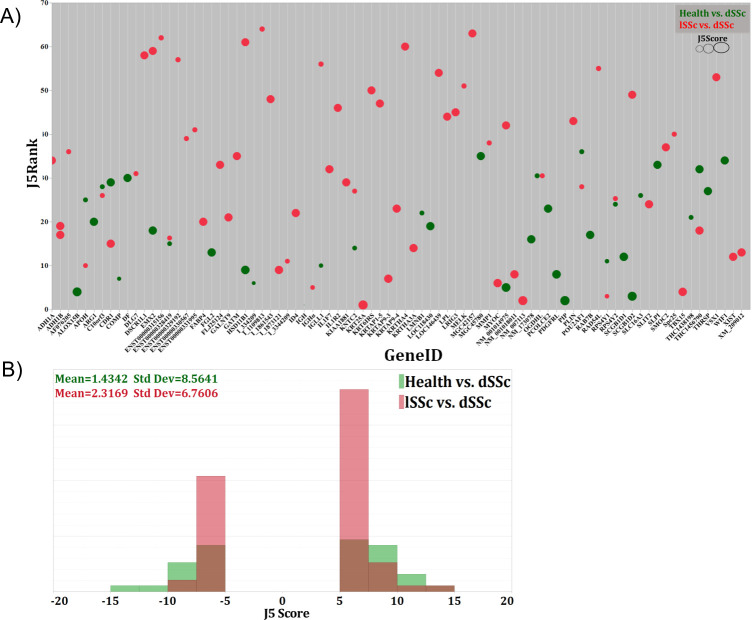
Qualitative and quantitative analysis contrasting disease types and related-gene expression pattern. Shown are (A) a bubble chart reflecting expression levels of statistically significant genes according to their J5-score and ranking and differentiating healthy vs dSSc as opposed to lSSc vs dSSc and, (B) the distribution of J5 scores contrasting healthy vs dSSc as opposed to lSSc vs dSSc.

We performed unsupervised analyses to compare the expression profiles from healthy and dSSc patients using a total of 54 biopsy samples. Using the J5 statistical test at a threshold of 7.0, we identified 36 genes that were considered differentially expressed between the groups (Fig 3A and S1 Fig). Among the differentially expressed genes were several [31] that are supported by the literature including *COMP* [4], *FGL2* [32], *WIF1* [2]. It was also evident that many matrix-related genes were differentially expressed between these two patient groups. We next tested the 36-gene list as a classifier index in a Naïve Bayes model to evaluate its ability to differentiate between genomic profiles of healthy patients from those with dSSc. Classification based upon expression of these genes was highly accurate, with 90% of samples being correctly categorized by the model, sensitivity of 0.871, and specificity of 1.0. We next compared the gene expression profiles of patients with lSSc to those with dSSc. This analysis used a total of 60 samples and, using a J5 threshold of 6.0, identified 64 genes that were significantly differentially expressed between the groups (Fig 3B and S2 Fig). As with the gene list that differentiated between normal and dSSc patients, we tested whether this 64-gene list could be used to classify patients with the two most common clinical subtypes of SSc: dSSc and lSSc. Classification using this panel of genes was accurate for 89% of samples, with sensitivity of 0.871 and specificity of 0.937. The overall theses analyses represent that there are gene expression patterns separating disease subtypes gene expression pattern of this panel is fundamentally heterogeneous. Although the average J5 score seems to be higher in lSSc vs dSSc as opposed to healthy vs dSSc, the gap in the overall levels of gene expression between dSSc and lSSc is reduced as reflected by the shift of both negative and positive J5 score towards the center in lSSc vs dSSc relative to healthy vs dSSc.

### Genomic profiles of healthy and dSSc patients differ in their expression of matrix and growth factor signaling genes

To learn more about the pathways and functional networks associated with these genes differentially expressed between healthy and dSSc patients, we performed pathway and impact analysis on the gene list. The pathways with the highest impact factors calculated by Pathway Express are shown in Table 1. Based on impact analysis score, the top three pathways identified were TGF-β signaling pathway, Wnt signaling pathway, and ECM-receptor interaction.

**Table 1:** Pathways associated with differentially expressed genes between healthy and dSSc patient biopsy samples.

Rank	Database Name	Pathway Name	Impact Factor	No. Genes in Pathway	No. Input Genes in Pathway	No. Pathway Genes on Chip	% Pathway Genes in Input	Corrected p-value	Sum (PF)	KEGG Pathway ID
1	KEGG	TGF-β signaling pathway	9.104	87	1	71	1.149	0.112591573	6.919863899	1:04350
2	KEGG	Wnt signaling pathway	6.415	152	1	123	0.658	0.18712644	4.738727096	1:04310
3	KEGG	ECM-receptor interaction	4.477	84	1	72	1.19	0.114085731	2.306564066	1:04512
4	KEGG	Primary immunodeficiency	4.446	35	1	21	2.857	0.034674686	1.084276803	1:05340
5	KEGG	Ribosome	3.255	101	1	71	0.99	0.112591573	1.071276617	1:03010
6	KEGG	Focal adhesion	2.563	203	1	166	0.493	0.244127768	1.153324958	1:04510

No. Genes in Pathway: Number of genes annotated for pathway, No. Input Genes in Pathway: Number of genes in input list that occur in pathway, No. Pathway Genes on Chip: Number of genes annotated for pathway for which there are probes on microarray chip, % Pathway Genes in Input: Percentage of genes that are annotated for pathway and included in input set, Corrected p-value: FDR-corrected p-value, Sum (PF): Sum of absolute values of perturbation factors.

We then performed pathway and impact analysis on the gene list differentially expressed between lSSc and dSSc patients. The top pathway associated with these differentially expressed genes was PPAR signaling with an associated impact factor of 11.982 and was statistically enriched by genes in our list (Table 2). Differentially expressed genes that were present in this pathway were *FABP4*, *LPL*, *MMP1*, and *PLIN*.

**Table 2:** Pathways associated with differentially expressed genes between lSSc and dSSc patient biopsy samples.

	Database Name	Pathway Name	Impact Factor	No. Genes in Pathway	No. Input Genes in Pathway	No. Pathway Genes on Chip	% Pathway Genes in Input	Corrected p-value	Sum (PF)	KEGG Pathway ID
1	KEGG	PPAR signaling pathway	11.982	70	4	52	5.714	1.67E-05	9.85E-01	1:03320
2	KEGG	Axon guidance	7.301	129	1	96	0.775	2.47E-01	5.90E+00	1:04360
3	KEGG	MAPK signaling pathway	4.294	272	1	217	0.368	4.75E-01	3.55E+00	1:04010
4	KEGG	Primary immunodeficiency	3.710	35	1	21	2.857	6.02E-02	8.99E-01	1:05340
5	KEGG	Homologous recombinant	3.585	28	1	24	3.571	6.84E-02	9.03E-01	1:03440
6	KEGG	Bladder cancer	3.265	42	1	36	2.381	1.01E-01	9.72E-01	1:05219
7	KEGG	Ribosome	2.905	101	1	71	0.99	1.89E-01	1.24E+00	1:03010
8	KEGG	TGF-β signaling pathway	2.700	87	1	71	1.149	1.89E-01	1.04E+00	1:04350
9	KEGG	Hematopoietic cell lineage	2.660	87	1	67	1.149	1.80E-01	9.43E-01	1:04640
10	KEGG	Alzheimer's disease	2.061	178	1	135	0.562	3.30E-01	9.51E-01	1:05010
11	KEGG	Cytokine-cytokind receptor interaction	1.857	263	1	173	0.38	4.01E-01	9.43E-01	1:04060
12	KEGG	Pathways in cancer	1.581	330	1	264	0.303	5.44E-01	9.72E-01	1:05200

No. Genes in Pathway: Number of genes annotated for pathway, No. Input Genes in Pathway: Number of genes in input list that occur in pathway, No. Pathway Genes on Chip: Number of genes annotated for pathway for which there are probes on microarray chip, % Pathway Genes in Input: Percentage of genes that are annotated for pathway and included in input set, Corrected p-value: FDR-corrected p-value, Sum (PF): Sum of absolute values of perturbation factors.

Next we use subset of genes with positive silhouette scores to expand the insights into the relationship between selected gene sets found to be differentially expressed between lSSc and dSSc patient biopsy samples based on J5 analysis by Enrichment analysis using PANTHER.

**Fig 4:**
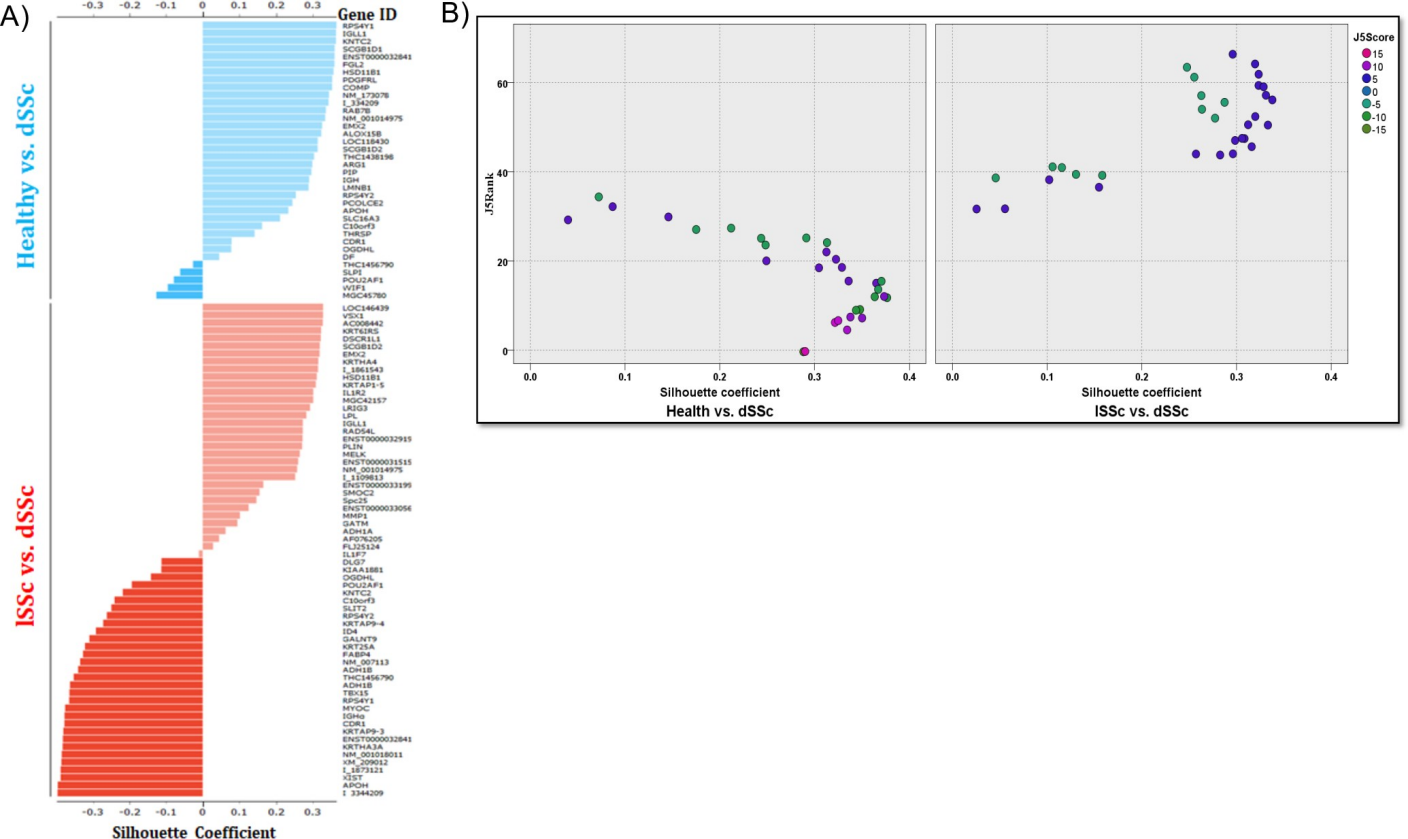
Silhouette analysis of genes differentially expressed between healthy and dSSc patient biopsy samples. (A) The silhouette analysis scores range from 1.0 to − 1.0, and a larger value for the average silhouette (AS) over all samples to be analyzed indicates a higher degree of cluster separation. Silhouette coefficients near +1 indicate that the feature is far away from the neighboring clusters. A value of 0 indicates that the sample is on or very close to the decision boundary between two neighboring clusters, and negative values indicate that those samples might have been assigned to the wrong cluster. (B) This scatterplot contrasting the positive silhouette scores healthy vs dSSc as opposed to lSSc.

This silhouette plot shows measure of how well a feature is clustered within a given cluster and the degree of separation from other clusters. A silhouette analysis of healthy vs. dSSc and lSSc vs. dSSc patients reveals distinct relationships between disease tight and differently expressed genes identified by J5 analysis (Fig 4A). Interestingly the scatterplot contrasting the positive silhouette scores healthy vs dSSc as opposed to lSSc shows a high degree of separation (Fig 4B).

**Fig 5:**
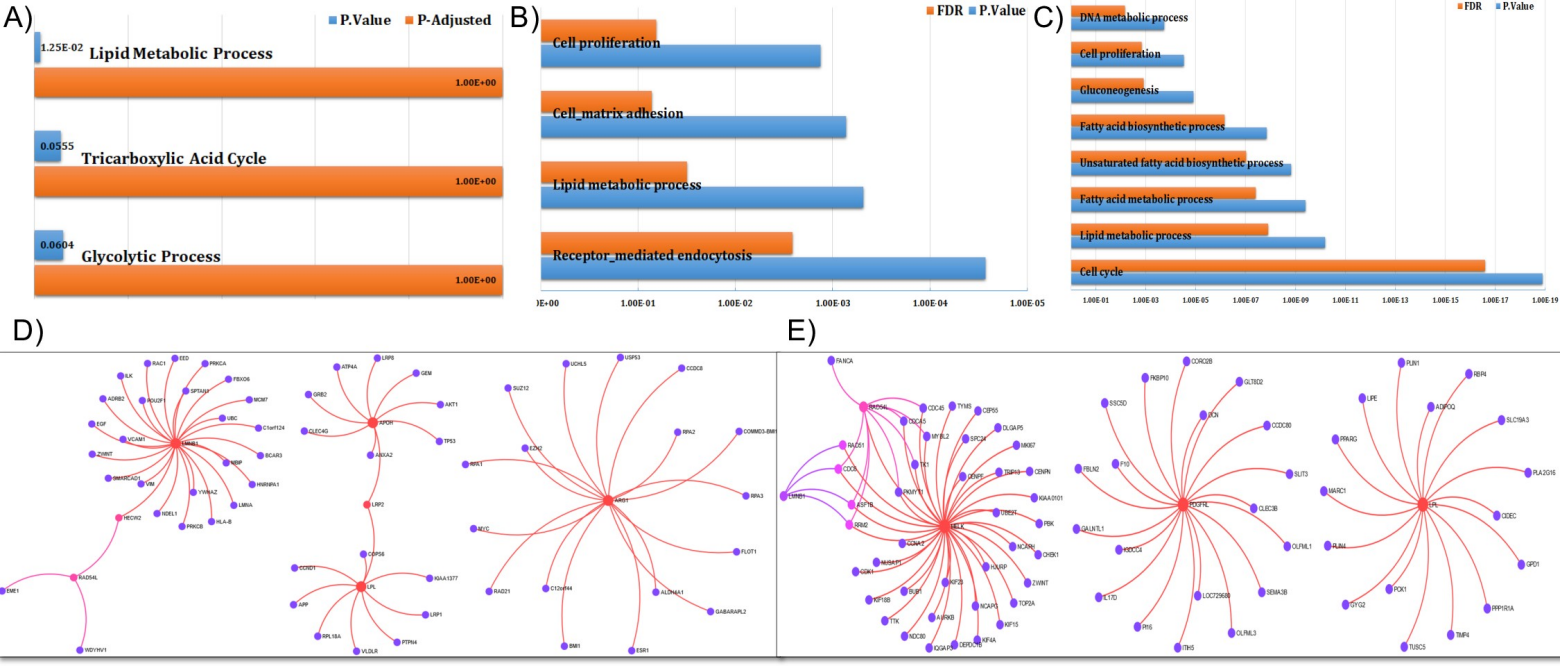
Enrichment analysis using PANTHER of genes differentially expressed between healthy and dSSc patient biopsy samples based on J5 analysis. (A) Enrichment analysis using PANTHER of the collective set of genes with positive silhouette scores (Protein Analysis Through Evolutionary Relationships, http://pantherdb.org). (B, D) Enrichment analysis of the collective set of genes with positive silhouette scores using PANTHER, based on the skin-specific protein-protein interactions, derived from the DifferentialNet database. (C, E) Enrichment analysis of the collective set of genes with positive silhouette scores using PANTHER, based on the skin-specific gene co-expression interactions, derived from the TCSBN database.

An enrichment analysis using PANTHER (Fig 5) of the collective set of genes with positive silhouette scores from the J5 analysis was used to analyze skin-specific protein-protein interaction. These analysis immune and extracellular matrix response and organization.

### Genes that differentiate lSSc and dSSc patients have a wide range of functions

**Fig 6:**
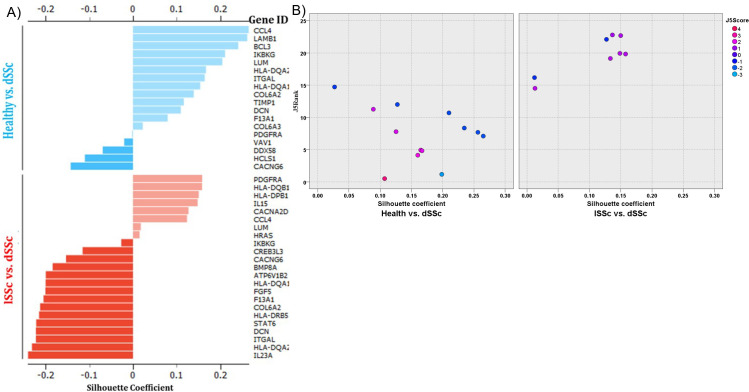
Silhouette analysis of genes differentially expressed between dSSc and lSSc patient biopsy samples. (A) The silhouette analysis scores range from 1.0 to − 1.0, and a larger value for the average silhouette (AS) over all samples to be analyzed indicates a higher degree of cluster separation. Silhouette coefficients near +1 indicate that the feature is far away from the neighboring clusters. A value of 0 indicates that the sample is on or very close to the decision boundary between two neighboring clusters, and negative values indicate that those samples might have been assigned to the wrong cluster. (B) This scatterplot contrasting the positive silhouette scores healthy vs dSSc as opposed to lSSc.

In contrast, the subset of genes with positive silhouette scores will be employed to gain more insights into the relationship between selected gene sets found to be differentially expressed between lSSc and dSSc patient biopsy samples based on J5 analysis by enrichment analysis using PANTHER [33] (Fig 6) shows a high degree of separation but the selected panel of genes/biomarkers correlates significantly with lipid metabolism.

### Mining and selection of genes to create predictive gene index (PDI)

Based on recent literature that shows a link between chemokine signaling and expression of extracellular matrix molecules, we tested our hypothesis that a curated list of immuno-modulatory and extracellular matrix genes is sufficient to predict disease severity or clinical subtype. We combined pathway- and literature-based methods to define our informed predictive gene index (PDI). We first searched for genes that appeared in pathways related to inflammation (8 pathways) and extracellular matrix (4 pathways), as defined by the Kyoto Encyclopedia of Genes and Genomes (KEGG) Database (www.kegg.jp) (Table 3). In addition, significant findings from literature mining led us to include the following genes: *TNC*, *DCN*, *FN1*, *COL1A2*, *TGFB*, *CXCR3*, and *CXCR4*. We chose a panel of 60 genes to use as our PDI, which served as the basis for our predictive modeling approach (Table 4).

**Table 3:** KEGG pathways used for selection of genes for predictive gene index (PDI). All pathways are Homo sapien.

	Pathway ID	Pathway Name
**Inflammation**	hsa04064	NF-κB signaling pathway
	hsa05321	Inflammatory bowel disease (IBD)
	hsa05323	Rheumatoid arthritis
	hsa04062	Chemokine signaling pathway
	hsa04668	TNF signaling pathway
	hsa04010	MAPK signaling pathway
	hsa04610	Complement and coagulation cascades
	hsa04066	HIF-1 signaling pathway
**Extracellular Matrix**	hsa04510	Focal adhesion
	hsa04350	TGF-β signaling pathway
	hsa04512	ECM-receptor interaction
	hsa05205	Proteoglycans in cancer

**Table 4:** 60 genes chosen for predictive gene index (PDI).

Gene Symbol	Gene Name	Associated Pathway [31]
TNC	TNC Tenascin	[KO:K05692] Focal adhesion, [KO:K06236] ECM-receptor interaction
DCN	DCN Decorin	[KO:K05692] Proteoglycans in cancer, [KO:K16622] TGF-β signaling pathway
FN1	FN1 Fibronectin 1	[KO:K05692] Focal adhesion, [KO:K05692] Proteoglycans in cancer, [KO:K06236] ECM-receptor interaction
COL1A2	COL1A2 Collagen type 1 alpha 2	[KO:K05692] Focal adhesion, [KO:K06236] ECM-receptor interaction
TGFB	TGFB1 Transforming Growth Factor, Beta 1	[KO:K04858] MAPK signaling pathway, [KO:K16622] TGF-β signaling pathway, [KO:K05692] Proteoglycans in cancer, [KO:K06752] Inflammatory bowel disease (IBD), [KO:K14624] Rheumatoid arthritis
CXCR3	CXCR3 C-X-C Chemokine Receptor Type 3	[KO:K05726] Chemokine signaling pathway
CXCR4	CXCR4 C-X-C Chemokine Receptor Type 4	[KO:K05726] Chemokine signaling pathway
A2M	A2M alpha-2-macroglobulin	[KO:K03910] Complement and coagulation cascades
ACTB	ACTB actin, beta	[KO:K05692] Focal adhesion, [KO:K05692] Proteoglycans in cancer
ATP6V1B2	ATP6V1B2 ATPase, H+ transporting, lysosomal 56/58kDa, V1 subunit B2	[KO:K02147] [EC:3.6.3.14] Rheumatoid arthritis
BCAR1	BCAR1 breast cancer anti-estrogen resistance 1	[KO:K05726] Chemokine signaling pathway, [KO:K05726] Focal adhesion
BCL3	BCL3 B-cell CLL/lymphoma 3	[KO:K09258] TNF signaling pathway
BMP8A	BMP8A bone morphogenetic protein 8a	[KO:K16622] TGF-β signaling pathway
CACNA2D1	CACNA2D1 calcium channel, voltage-dependent, alpha 2/delta subunit 1	[KO:K04858] MAPK signaling pathway
CACNG6	CACNG6 calcium channel, voltage-dependent, gamma subunit 6	[KO:K04871] MAPK signaling pathway
CAV2	CAV2 caveolin 2	[KO:K12958] Focal adhesion, [KO:K12958] Proteoglycans in cancer
CCL2	CCL2 C-C motif chemokine ligand 2	[KO:K14624] TNF signaling pathway, [KO:K14624] Rheumatoid arthritis, [KO:K14624] Chemokine signaling
CCL4	CCL4 C-C motif chemokine ligand 4	[KO:K12964] NF-κB signaling, [KO:K12964] Chemokine signaling pathway
CCR5	CCR5 C-C motif chemokine receptor 5 (gene/pseudogene)	[KO:K04180] Chemokine signaling pathway
CD86	CD86 CD86 molecule	[KO:K05413] Rheumatoid arthritis
COL1A2	COL1A2 collagen, type I, alpha 2	[KO:K06236] Focal adhesion, [KO:K06236] ECM-receptor interaction
COL6A2	COL6A2 collagen, type VI, alpha 2	[KO:K06238] Focal adhesion, [KO:K06238] ECM-receptor interaction
COL6A3	COL6A3 collagen, type VI, alpha 3	[KO:K06238] Focal adhesion, [KO:K06238] ECM-receptor interaction
CREB3L3	CREB3L3 cAMP responsive element binding protein 3-like 3	[KO:K09048] TNF signaling pathway
CXCL5	CXCL5 chemokine (C-X-C motif) ligand 5	[KO:K05506] Rheumatoid arthritis, [KO:K05506] Chemokine signaling, [KO:K05506] TNF signaling pathway
DDX58	DDX58 DEAD (Asp-Glu-Ala-Asp) box polypeptide 58	[KO:K12646] [EC:3.6.3.14] NF-κB B signaling pathway
EIF4B	EIF4B eukaryotic translation initiation factor 4B	[KO:K03258] Proteoglycans in cancer
F13A1	F13A1 coagulation factor XIII, A1 polypeptide	[KO:K03917] [EC:2.3.2.13] Complement and coagulation cascades
F7	F7 coagulation factor VII (serum prothrombin conversion accelerator)	[KO:K01320] [EC:3.4.21.21] Complement and coagulation cascades
FGF19	FGF19 fibroblast growth factor 19	[KO:K04358] MAPK signaling pathway, [KO:K04358] Proteoglycans in cancer
FGF5	FGF5 fibroblast growth factor 5	[KO:K04358] MAPK signaling pathway, [KO:K04358] Proteoglycans in cancer
HCLS1	HCLS1 hematopoietic cell-specific Lyn substrate 1	[KO:K06106]Proteoglycans in cancer
HLA-DMA	HLA-DMA major histocompatibility complex, class II, DM alpha	[KO:K06752]Inflammatory bowel disease (IBD), [KO:K06752] Rheumatoid arthritis
HLA-DOA	HLA-DOA major histocompatibility complex, class II, DO alpha	[KO:K06752]Inflammatory bowel disease (IBD), alpha [KO:K06752] Rheumatoid arthritis
HLA-DPA1	HLA-DPA1 major histocompatibility complex, class II, DP alpha 1 [	KO:K06752] Inflammatory bowel disease (IBD), [KO:K06752] Rheumatoid arthritis
HLA-DPB1	HLA-DPB1 major histocompatibility complex, class II, DP beta 1	[KO:K06752] Inflammatory bowel disease (IBD), [KO:K06752] Rheumatoid arthritis
HLA-DQA1	HLA-DQA1 major histocompatibility complex, class II, DQ alpha 1	[KO:K06752] Inflammatory bowel disease (IBD), [KO:K06752] Rheumatoid arthritis
HLA-DQA2	HLA-DQA2 major histocompatibility complex, class II, DQ alpha 2	[KO:K06752] Inflammatory bowel disease (IBD), [KO:K06752] Rheumatoid arthritis
HLA-DQB1	HLA-DQB1 major histocompatibility complex, class II, DQ beta 1	[KO:K06752] Inflammatory bowel disease (IBD), [KO:K06752] Rheumatoid arthritis
HLA-DRB5	HLA-DRB5 major histocompatibility complex, class II, DR beta 5	[KO:K06752] Inflammatory bowel disease (IBD), [KO:K06752] Rheumatoid arthritis
HRAS	HRAS Harvey rat sarcoma viral oncogene homolog	[KO:K02833] Chemokine signaling pathway, [KO:K02833] MAPK signaling
		Pathway, [KO:K02833] Focal adhesion, [KO:K02833] Proteoglycans in cancer
IKBKG	IKBKG inhibitor of kappa light polypeptide gene enhancer in B-cells, kinase gamma	[KO:K07210] MAPK signaling pathway, [KO:K07210] NF-κB signaling pathway, [KO:K07210] Chemokine signaling pathway, [KO:K07210] TNF signaling pathway
IL15	IL15 interleukin 15	[KO:K05433] TNF signaling pathway, [KO:K05433] Rheumatoid arthritis
IL23A	IL23A interleukin 23, alpha subunit p19	[KO:K05426] Inflammatory bowel disease (IBD), [KO:K05426] Rheumatoid arthritis
ITGAL	ITGAL integrin, alpha L (antigen CD11A (p180), lymphocyte function-associated antigen 1	[KO:K05718] Rheumatoid arthritis
ITGB1	ITGB1 integrin, beta 1 (fibronectin receptor, beta polypeptide, antigen CD29 includes MDF2, MSK12)	[KO:K05719] ECM-receptor interaction, [KO:K05719] Focal adhesion, [KO:K05719] Proteoglycans in cancer
ITGB2	ITGB2 integrin, beta 2 (complement component 3 receptor 3 and 4 subunit)	[KO:K06464] Rheumatoid arthritis
LAMB1	LAMB1 laminin, beta 1	[KO:K05636] Focal adhesion, [KO:K05636] ECM-receptor interaction
LUM	LUM lumican	[KO:K08122] Proteoglycans in cancer
MSN	MSN moesin	[KO:K05763] Proteoglycans in canceR
PDGFC	PDGFC platelet derived growth factor C	[KO:K05450] Focal adhesion
PDGFRA	PDGFRA platelet-derived growth factor receptor, alpha polypeptide	[KO:K04363] [EC:2.7.10.1] MAPK signaling pathway, [KO:K04363] [EC:2.7.10.1] Focal adhesion
PLAUR	PLAU plasminogen activator, urokinase	[KO:K01348] [EC:3.4.21.73] Proteoglycans in cancer, [KO:K01348] [EC:3.4.21.73] NF-κB signaling pathway, [KO:K01348] [EC:3.4.21.73] Complement and coagulation cascades
RAC2	RAC2 ras-related C3 botulinum toxin substrate 2 (rho family, small GTP binding protein Rac2)	[KO:K07860] Focal adhesion, [KO:K07860] Chemokine signaling pathway, [KO:K07860] MAPK signaling pathway
SMAD1	SMAD1 SMAD family member 1	[KO:K04676] TGF-β signaling pathway
SP1	SP1 Sp1 transcription factor	[KO:K04684] TGF-β signaling pathway
STAT6	STAT6 signal transducer and activator of transcription 6, interleukin-4 induced	[KO:K11225] Inflammatory bowel disease (IBD)
TGFBR2	TGFBR2 transforming growth factor, beta receptor II (70/80kDa)	[KO:K04388] [EC:2.7.11.30] TGF-β signaling pathway, [KO:K04388] [EC:2.7.11.30] MAPK signaling pathway
TIMP1	TIMP1 TIMP metallopeptidase inhibitor 1	[KO:K16451] HIF-1 signaling pathway
VAV1	VAV1 vav 1 guanine nucleotide exchange factor	[KO:K05730] Chemokine signaling pathway, [KO:K05730] Focal adhesion

### Inflammation and ECM based Naïve Bayes classification algorithm accurately distinguishes between patient gene expression profiles

We next assessed the ability of our 60-gene PDI to distinguish between gene profiles from healthy and dSSc patient samples, based on gene profile data alone. Using a J5 threshold of 1.4, 18 of the genes from our PDI were identified as being differentially expressed between the healthy and dSSc groups. Among the most significant genes were *DCN* and *LUM* (Table 5). PACE analysis indicated that the Naïve Bayes model was significant at PACE 0.045 to J5 1.4 (S3 Fig). The model achieved sensitivity of 0.948 and specificity of 1.0. We also assessed whether our model could accurately differentiate between patients with lSSc and dSSc. When comparing between disease subtype, using J5 threshold of 1.4, 23 genes were differentially expressed, with many being related to major histone compatibility complex (MHC) genes (Table 6). For this comparison, the Naïve Bayes model was significant at PACE 0.05 to J5 1.1 (S4 Fig). The model achieved sensitivity of 0.665 and specificity of 0.814. Lastly, to streamline the predictive gene index, we selected the genes that had the best predictive power to differentiate between high or low severity and among disease subsets, resulting in a final 12-gene index-based classifier that could accurately predict patient outcome based on gene expression profiles from patient skin biopsies (Fig 8). The genes comprising the 12-gene index were *PDGFRA*, *BMP8A*, *IL15*, *CXCL5*, *STAT6*, *F13A1*, *CACNG3*, *ITGAL*, *COL6A2*, *HLA-DQA1*, *HLA-DQB1*, and *HLA-DRB5*.

**Fig 7:**
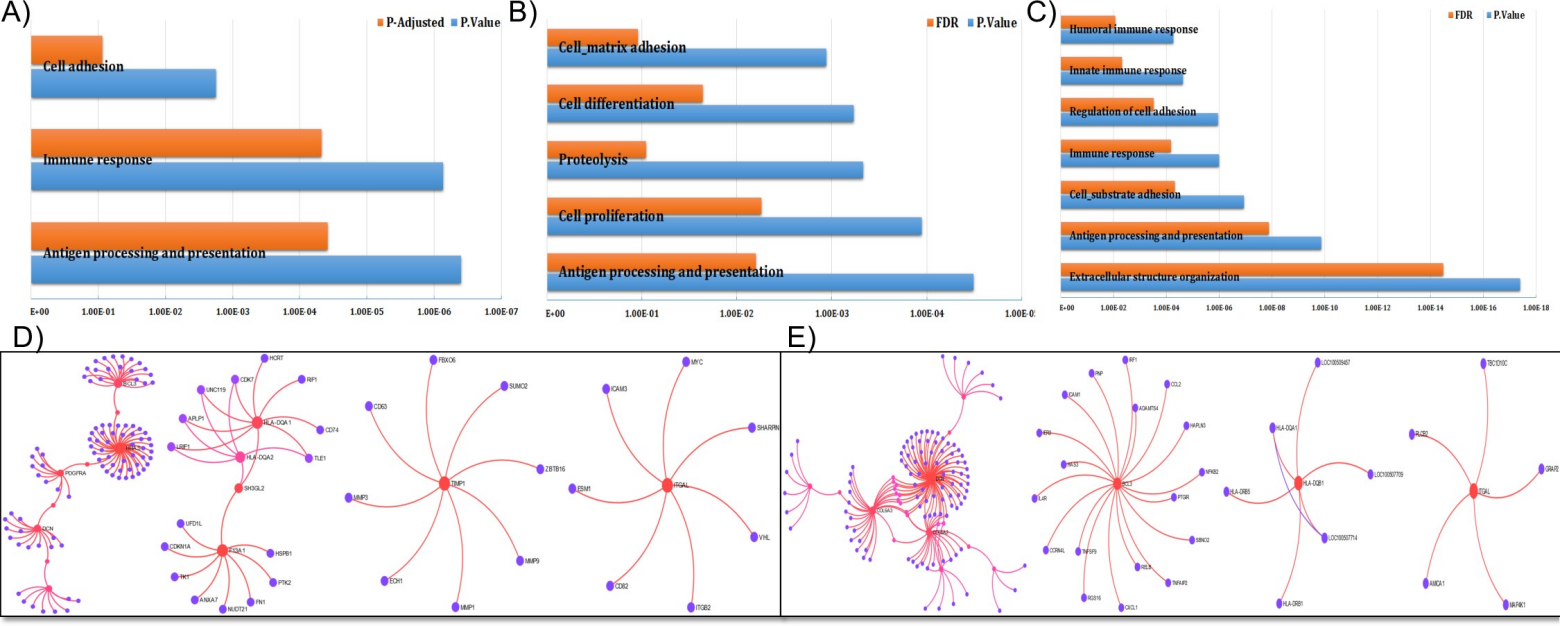
Enrichment analysis using PANTHER of genes differentially expressed between lSSc and dSSc patient biopsy samples based on J5 analysis. (A) Enrichment analysis using PANTHER of the collective set of genes with positive silhouette scores (Protein Analysis Through Evolutionary Relationships, http://pantherdb.org). (B, D) Enrichment analysis of the collective set of genes with positive silhouette scores using PANTHER, based on the skin-specific protein-protein interactions, derived from the DifferentialNet database. (C, E) Enrichment analysis of the collective set of genes with positive silhouette scores using PANTHER, based on the skin-specific gene co-expression interactions, derived from the TCSBN database.

**Fig 8:**
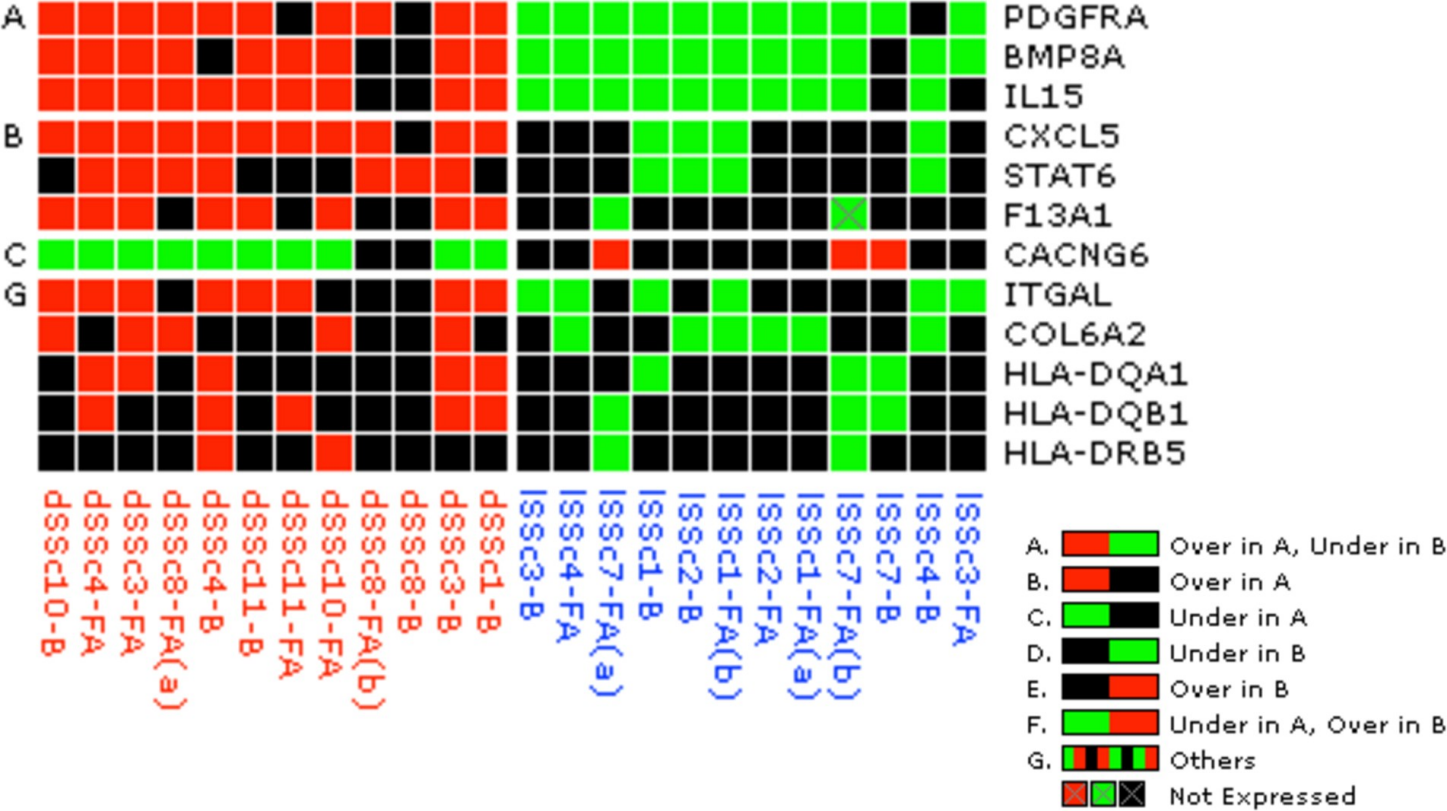
Gene expression grid showing expression of genes in 12-gene panel capable of predicting disease features. Color of boxes indicates directionality of expression differences with red indicating high expression and green indicating low expression. Patient samples highlighted in red were all from dSSc patients and were higher severity (mean mRSS 35.6); samples highlighted in blue were all from lSSc patients and were lower severity (mean mRSS 7.73).

**Table 5:** Genes from predictive gene index that were differentially expressed between healthy control and dSSc patient biopsy samples.

J5 Rank	Gene ID	J5 Score
1	DCN	3.552
2	LUM	-2.729
3	HLA-DQA1	2.198
4	ITGAL	2.067
5	HLA-DQA2	1.907
6	LAMB1	-1.814
7	CCL4	-1.766
8	COL6A2	1.738
9	BCL3	-1.725
10	IKBKG	-1.723
11	F13A1	1.635
12	TIMP1	-1.621
13	PDGFRA	1.599
14	COL6A3	-1.496
15	VAV1	-1.487
16	DDX58	-1.467
17	HCLS1	-1.447
18	CACNG6	-1.405

**Table 6:** Genes from predictive gene index that were differentially expressed between lSSc and dSSc patient biopsy samples.

J5 Rank	Gene ID	J5 Score
1	HLA-DQA1	3.11
2	F13A1	3.014
3	HLA-DRB5	2.812
4	STAT6	2.679
5	HLA-DQA2	2.296
6	ITGAL	2.236
7	DCN	1.94
8	COL6A2	1.93
9	ATP6V1B2	1.729
10	BMP8A	1.622
11	IL23A	-1.572
12	FGF5	-1.561
13	CACNG6	-1.522
14	CREB3L3	-1.441
15	HRAS	1.426
16	IKBKG	-1.397
17	LUM	-1.372
18	CACNA2D1	1.37
69	IL15	1.345
20	HLA-DQB1	1.336
21	CCL4	-1.307
22	PDGFRA	1.245
23	HLA-DPB1	1.109

## Discussion

Fibrotic diseases, including systemic sclerosis (scleroderma, SSc), remain debilitating, costly, and painful conditions for thousands of patients. Current treatment strategies often fail in segments of the patient population [34]. These failures have largely been attributed to heterogeneity of disease presentation and progression. In addition, current animal models do not capture the full spectrum of gene expression that underlies various subtypes of human disease [35].

In the absence of definitive biomarkers of SSc pathogenesis, mRSS scores may be confounded by the natural history of disease with age, making comparisons across age groups convoluted (Fig 1). Demographic data analysis has revealed age, but not race, gender and skin origin (Fig 2) to be reliable predictors of SSc disease subtype through a Bayesian network and max-min hill climbing (MMHC) structured learning algorithm (Fig 2A) [36]. Linear projection modeling revealed various ages amongst dSSc patients included in this study, but lSSc patients were found to be older with a narrow range in skin scores (Fig 2B). A study of 67 SSc patients by Perez-Bocanegra et al. also found a likelihood of the lSSc subtype in older patients as well as increased occurrence and more rapid onset of cardiac and pulmonary symptons with age [37]. More investigation into age and SSc subtype may stand as both a promising diagnostic tool and insight into divergent disease subtype development.

Previous studies have used modeling approaches to identify important biomarker genes and classify SSc patients in a more robust manner than with clinical measurements alone [3, 4]. More recently, investigators have focused on panels comprising a handful of biomarkers to predict disease severity based on gene expression profiling [2, 5]. However, there have been no investigations that focused on the correlation between levels of chemokine and inflammation genes, which are known to be perturbed in disease [38, 39], and the expression levels of ECM genes. Therefore, in the present study we sought to identify the inflammation and ECM genes that were most important in predicting patient severity or disease subset, using SSc as a prototype of fibrotic disease.

To meet this goal, we used both unsupervised and literature-based methods to identify gene signatures that could distinguish healthy controls from dSSc patients and dSSc patients from lSSc patients. Our unsupervised, J5-based method revealed several genes that were differentially expressed between healthy and dSSc patients (Fig 3, S1 Fig). In several cases, our methodology confirmed associations that had previously been noted. We found Wnt signaling, TGF-β signaling, and ECM associated genes to be upregulated (Table 1), which has been confirmed at the mRNA and miRNA level in SSc fibroblasts [40]. The Wnt/β -catenin signaling pathway is over activated in SSc patients and expression of *WIF1*, a Wnt pathway antagonist, is decreased in SSc patients [41], likely through a reactive oxygen species-dependent transcriptional repression mechanism [42]. WIF1 has been posed as part of a biomarker panel for the prediction of skin involvement in dSSc [2]. Therefore, we were not surprised to find that our J5 analysis showed *WIF1* was differentially expressed between expression profiles of healthy and dSSc patients. Our analysis also highlighted ECM protein cartilage oligomeric matrix protein (*COMP*) (Fig 4, S1 Fig), a gene that is overexpressed in skin of SSc patients [43]. Serum concentration of *COMP* is associated with mortality risk in SSc patients and it is one gene in a four gene biomarker panel proposed by Farina et al. for assessing the severity of dSSc [4, 5]. We also found that expression of fibrinogen-like protein 2 (*FGL2*), a glycoprotein that is increased in serum of SSc patients [32], was different between healthy and dSSc patients (Fig 4, S1 Fig). Further analysis showed that the genes characterizing healthy or dSSc profiles were ranked as having high impact on pathways that are critical to the pathogenesis of fibrosis, including TGF-β signaling, Wnt signaling, ECM-receptor interaction, and immunodeficiency [44–46]. Along with these genes, our analysis allowed us to identify several genes that warrant further investigation, including genes related to immune response (*IGH*, *ALOX15B*), growth factor signaling (*PDGFRL*), and extracellular matrix adhesion (*LMNB1*) (Fig 4, S1 Fig).

Limited (lSSc) and diffuse (dSSc) scleroderma are clinically defined subtypes that differ in both clinical presentation and in terms of which organs are most commonly affected by disease. Patients with dSSc have severe skin involvement, which often rapidly spreads across the body and frequently have cardiac and renal involvement and interstitial lung disease [47, 48]. While skin involvement in lSSc patients is usually confined to the hands and face, these patients are more likely to develop pulmonary arterial hypertension than dSSc patients [49]. In the context of gene expression, previous studies have shown subset-level differences in DNA methylation patterns [50], TGF-β signaling [51], and immune response genes [52] between dSSc and lSSc patients, particularly in fibroblastic gene signatures, the cell type primarily responsible for matrix production [53]. Our J5 analysis identified several genes that were differentially expressed between these disease subtypes (Fig 3, S2 Fig). Matrix metalloproteinases (MMPs) are known to play a central role in fibrosis through their ability break down ECM constituents. Recent studies have also suggested a role for MMP upregulation in sustained inflammation through the immune cells chemoattraction and proliferation [54, 55], particularly in older individuals [56], suggesting a role of MMP’s in the highly interdependent age and skin score correlations revealed through our Bayesian network projections (Fig 2). Along with several other MMPs and their inhibitors, levels of MMP-1 show close association with SSc, and we found that gene expression differed between dSSc and lSSc patient profiles (Fig 6). Serum levels of MMPs are increased in a subset of patients [57], polymorphisms are associated with various clinical features of disease [58], and anti-MMP1 antibodies are elevated in lSSc patients [59]. To our knowledge, this is the first study indicating that transcript levels of *MMP1* may differ between lSSc and dSSc patients. Interestingly, an earlier study showed that serum levels of MMP9 were significantly higher in dSSc than lSSc patients [60]. Hence, further investigation may show MMPs to be a diagnostic marker of SSc disease subtype beyond that of SSc at large.

We also found that lipoprotein lipase (*LPL*) was differentially expressed between disease subtypes (Fig 6, S2 Fig). A 2005 study found that antibodies against LPL were present in about a third of SSc patients and were associated with organ involvement. Interestingly, the authors found no difference in levels of anti-LPL between dSSc and lSSc patients [61]. Based on the evidence presented within the literature [62], our selected panel of genes/biomarkers differentiating lSSc and dSSc patients correlates significantly with lipid metabolism (Fig 7) which could lead to a minimally invasive means for early detection and monitoring of disease [63, 64].

Similar to the analysis of healthy controls and dSSc patients, our comparison of gene profiles between patients with dSSc and lSSc revealed several novel, potential biomarkers that might be of interest for future study. Our pathway analysis showed PPAR signaling (Table 1) as a top pathway associated with genes expressed between disease subsets. Recent work shows that levels of PPAR-γ, which can antagonize TGF-β signaling, are low and dysregulated in patients with SSc [65, 66].

Classification models built using these differentially expressed genes were highly accurate in discerning between severity of disease or disease subtype, indicating that our methods identified panels of genes that were highly correlated with clinical features of interest. However, these gene lists were not rooted in known associations with disease that link to mechanisms of inflammation and extracellular matrix production. Instead of relying on a completely nonparametric approach, we aimed to develop a gene signature that would meaningfully relate to what is known about the development of fibrotic diseases. Based on the pathways identified in the first analyses, we used the available literature to hone in on categories central to the pathogenesis of SSc, extracellular matrix production and inflammation, and mined the literature and known pathways to develop our predictive gene index (PDI). We included specific chemokines and receptors that have been tied to fibrotic diseases, including *CXCL3*, *CXCL4*, *CCL2*, and *CCR5* and extracellular matrix molecules that are known to relate to disease such as *COL1A2* and *LUM* [67–70]. Together, our study underscores the importance of the 60 genes (and associated pathways) that we chose in differentiating between healthy and disease, and disease subsets. While it is known that modulation of the ECM and inflammation are key to the development of fibrosis, it was unclear which genes were most closely associated with progression of disease or which defined disease subtypes. The subset of our 60 genes that were differentially expressed between groups were highly accurate in discerning between different conditions when applied to a Naïve Bayes model, indicating that the regulation of these inflammatory and ECM genes may be closely tied to disease pathology. Thus, the ability of our model to faithfully predict severity based on these genes highlights their importance in disease pathogenesis and sheds light on this important aspect of SSc research. Our 12-gene panel represents the genes that might be of the highest relevance to distinguishing between disease states (Fig 8), when considered together.

Furthermore, genes from the predictive gene index identified herein may represent those that should be investigated to develop more clinically representative animal models for therapeutic testing. Recent work has highlighted the fact that murine models commonly used to study SSc do not capture the heterogeneity of human disease [35]. Single gene mutations and knockouts are not sufficient to recapitulate the unique, complex nature of SSc, which leads to poor understanding of disease and therapeutic efficacy. We propose that identification of a gene signature associated with SSc can be considered when developing small animal models with multiple mutations.

The utility of this PDI could be increased if it would be used to predict changes in severity. A longitudinal study would inform whether this model could be used as a prognostic indicator. Furthermore, some lSSc patients progress into dSSc with time. This parallels our findings that the overall discrepancies in gene expression level between dSSc and lSSc skin biopsies is reduced as reflected by the shift of both negative and positive J5 score towards the center in lSSc vs dSSc relative to healthy vs dSSc (Fig 3). A longitudinal study could also be used to evaluate whether any of the “incorrect” prediction classifications from our model that distinguishes lSSc patients from dSSc patients would actually be correct over time and provide insight into those mechanisms of disease progression that currently go undetected.

Another extension of this model is to include other clinical features to stratify patients by characteristics such as organ involvement, autoantibody profile, or to evaluate efficacy of treatments. Future research should investigate the biological mechanisms by which these chemokines and receptors function to modulate production and/or turnover of ECM constituents in disease.

## Methods

### Data retrieval

Whole-genome DNA microarrays were performed on skin biopsies taken from 34 individuals: 27 from distinct SSc subsets, and 6 healthy controls were used. Sixty-one skin biopsies (multiple biopsies per patient in some cases) and 14 technical replicates were analyzed, resulting in a total of 75 microarray hybridizations. All 75 microarray experiments were included. Skin biopsies were taken from the forearm or lower back. All data are publicly available at the National Center for Biotechnology Information GEO database (http://www.ncbi.nlm.nih.gov/geo; Accession Number: GSE9285) and were originally reported by Milano et al. [29].

### Efficiency analysis

Median, raw-intensity, expression values were formatted and annotated by the GPCL-Bioinformatic Analysis Core. Methods for normalization and identification of differentially expressed genes were evaluated using the objective function of maximum internal consistency using efficiency analysis (measured as the consistency in finding the method, including normalization, test and threshold, with the most reproducible set of retained genes during split dataset perturbations). The optimal cut off was selected as the maximum peak of internal consistency at overlap (0 < N3 < N max). The optimized methods for the two comparisons were then applied to the entire data set for each comparison using caGEDA [28]. False discovery rate estimation was conducted using a two-step method [71]. Differentially expressed genes were identified by efficiency analysis (EA), which finds the optimal combination of normalization, transformation, and feature selection techniques to find the most internally consistent set of differentially expressed genes, using AutoEA software [72].

### Tests for differential expression

Data transformation and normalization were optimized using efficiency analysis among and between groups. In all comparisons, differentially expressed genes were identified using the J5 test, which is a gene-specific ratio that compares the mean difference in expression intensity between two groups that are being compared to the average mean group difference of all genes in the array. The J5 score was calculated by dividing the mean difference between comparative by the average absolute mean difference of all genes in the data set. Its sign indicates the directionality.

$${J5}_{i}=\frac{{\bar{A}}_{i}-{\bar{B}}_{i}}{\frac{1}{m}\sum_{j=1}^{m}\left|{\bar{A}}_{j}-{\bar{B}}_{j}\right|}$$

This test is especially useful in cases where there are no accurate estimates of variance, when T-tests are likely to produce high false discovery rates. Analyses were performed using the caGEDA software [28].

### Computational prediction

A stringent method was used to explore genes that correlate with the mRSS. Various types of cross-validation, and optimized prediction modeling were undertaken; feature selection (identifying differentially expressed genes) was appropriately nested within the cross-validation loop. Multiple splits between training and test sets were used to minimize stochastic performance due to particular splits. Alternative methods for transformation and normalization were explored using the caGEDA software [28]. Specific classes of prediction modeling algorithms included Naïve Bayes, logistic regression, random forests, and a genetic-algorithm k of m model in which the model is optimized toward a weighted, achieved classification error. Results were validated using Permutation Achieved Classification Error (PACE) analysis [73], a technique which uses permutations of the dataset to assess the statistical significance of each prediction models’ achieved classification errors at given levels. PACE performance statistic of the classifier on true data samples and validates the consistent behavior of the classifier on the same data with randomly reassigned class labels. PACE analysis was use to assess significance of classification results we achieved from published data sets.

Summary scores were generated for each patient based on expression of the genes in our 60-gene predictive gene index. The sum of squared differences for the gene panel was used to rank all samples from high to low. Cut points for classifying new samples in groups along the index were derived based on the accuracy of the resulting classification rules and was evaluated using internal cross-validation. The final reduced set of 12 genes was evaluated as an index-based classifier.

### Functional analysis

Probe identifications and fold-change values for differentially expressed genes were then submitted to Pathway Express (Onto-Tools, Detroit, MI) for impact analysis [74] and further investigation of known genes, molecular networks, biological pathways, and functions. Impact analysis uses a hypergeometric test to identify canonical pathways that are significantly overrepresented in the list of differentially expressed genes compared to their expected representativeness, given the complement of genes on the original microarray, using KEGG pathways as a reference [75]. The iPLEX (San Diego, CA) genotype data analysis was conducted to find an association with the outcome using the Fisher exact test. Further analysis of the differentially expressed genes was conducted with open-access online bioinformatics tools (e.g., DAVID, Frederick, MD) [76] and programs licensed by the University of Pittsburgh Health Sciences Library (e.g., GeneSpring, Agilent technologies, Santa Clara, CA) for cross-referencing and data mining purposes. The pathways and networks identified in Ingenuity Pathway Analysis (IPA) (Qiagen) were used to guide interpretation of the potential function of the differentially expressed genes in relation to the biology of the microarray analyses.

All visualizations were made using R (cran.r-project.org) or Python (www.python.org) programming languages.

## Supporting information

S1 Table Descriptive statistics of skin donor biopsy score as function of the donor demographics. (DOCX)

S2 Table Bayesian network model conditional probabilities of disease type. (DOCX)

S1 Fig Gene expression grid showing expression of genes identified by J5 analysis as differentially expressed between genomic profiles of healthy controls and dSSc patient biopsy samples. Color of boxes indicates directionality of expression differences with red indicating high expression and green indicating low expression.
(TIF)

S2 Fig Gene expression grid showing expression of genes identified by J5 analysis as differentially expressed between genomic profiles of dSSc and lSSc patient biopsy samples. Color of boxes indicates directionality of expression differences with red indicating high expression and green indicating low expression.
(TIF)

S3 Fig PACE analysis of Naïve Bayes model for classification of genomic profiles from healthy control compared to dSSc patient biopsy samples. The model was significant at PACE 0.045 up to J5 1.4.
(TIF)

S4 Fig PACE analysis of Naïve Bayes model for classification of genomic profiles from lSSc compared to dSSc patient biopsy samples. The model was significant at PACE 0.05 up to J5 1.1.
(TIF)
