# Supplementary material for: Prediction of severity and subtype of fibrosing disease using model informed by inflammation and extracellular matrix gene index
Source: PLoS One. 2020 Oct 23;15(10):e0240986. doi: 10.1371/journal.pone.0240986 (PMC7584227; doi:10.1371/journal.pone.0240986)
Supplement: S1 Table — (DOCX) [file pone.0240986.s001.docx]

S1 Table

|  | Skin Score Statistics by Disease Type | | | | | | | | | | | |
| --- | --- | --- | --- | --- | --- | --- | --- | --- | --- | --- | --- | --- |
|  | dSSc | | | | lSSc | | | | Nor/Mor/EF | | | |
| Gender | Mean | Std Dev | Variance | Median | Mean | Std Dev | Variance | Median | Mean | Std Dev | Variance | Median |
| F | 23.384615384615 | 8.602689446489 | 74.006153846154 | 23 | 8 | 0.9607689228305 | 0.92307 69230769 | 8 | 0 | 0 | 0 | 0 |
| M | 26.583333333333 | 11.065575886368 | 122.44696969697 | 26 | 8 | 0 | 0 | 8 | 0 | 0 | 0 | 0 |

|  | Skin Score Statistics by Disease Type | | | | | | | | | | | |
| --- | --- | --- | --- | --- | --- | --- | --- | --- | --- | --- | --- | --- |
|  | dSSc | | | | lSSc | | | | Nor/Mor/EF | | | |
| Race | Mean | Std Dev | Variance | Median | Mean | Std Dev | Variance | Median | Mean | Std Dev | Variance | Median |
| A | 37.25 | 11.5 | 132.25 | 43 | - | - | - | - | - | - | - | - |
| AA | 35 | 0 | 0 | 35 | 9 | 0 | 0 | 9 | - | - | - | - |
| H | 35 | 0 | 0 | 35 | - | - | - | - | - | - | - | - |
| W | 21.266666666667 | 7.1435204946491 | 51.029885057471 | 22 | 7.8571428571429 | 0.8644378215076 | 0.74725 27472527 | 8 | - | - | - | - |

|  | Skin Score Statistics by Disease Type | | | | | | | | | | | |
| --- | --- | --- | --- | --- | --- | --- | --- | --- | --- | --- | --- | --- |
|  | dSSc | | | | lSSc | | | | Nor/Mor/EF | | | |
| Biopsy Origin | Mean | Std Dev | Variance | Median | Mean | Std Dev | Variance | Median | Mean | Std Dev | Variance | Median |
| Back | 23.941176470588 | 9.5882127390568 | 91.933823529412 | 26 | 8 | 1 | 1 | 8 | 0 | 0 | 0 | 0 |
| ForeArm | 24.761904761905 | 9.4968666511895 | 90.190476190476 | 26 | 8 | 0.8660254037844 | 0.75 | 8 | 0 | 0 | 0 | 0 |
| Morphea | - | - | - | - | - | - | - | - | 0 | 0 | 0 | 0 |
