# Supplementary material for: Prediction of severity and subtype of fibrosing disease using model informed by inflammation and extracellular matrix gene index
Source: PLoS One. 2020 Oct 23;15(10):e0240986. doi: 10.1371/journal.pone.0240986 (PMC7584227; doi:10.1371/journal.pone.0240986)
Supplement: S2 Table — (DOCX) [file pone.0240986.s002.docx]

S2 Table

| Conditional Probabilities of Disease Type | | | | | | | | |
| --- | --- | --- | --- | --- | --- | --- | --- | --- |
| Biopsy Origin | Age | Race | Skin Score | EF | Nor | dSSc | lSSc | Morph |
| Back | <= 34.2 | H | > 34.4 | 0.01 | 0.01 | 0.96 | 0.01 | 0.01 |
| Back | <= 34.2 | W | <= 8.6 | 0.01 | 0.98 | 0.0l | 0.01 | 0.01 |
| Back | 34.2~42.4 | AA | > 34.4 | 0.01 | 0.01 | 0.96 | 0.01 | 0.01 |
| Back | 34.2~42.4 | W | <= 8.6 | 0.01 | 0.49 | 0.01 | 0.49 | 0.01 |
| Back | 34.2~42.4 | W | 25.8~34.4 | 0.01 | 0.01 | 0.96 | 0.01 | 0.01 |
| Back | 42.4~50.6 | W | <= 8.6 | 0.01 | 0.98 | 0.01 | 0.01 | 0.01 |
| Back | 42.4~50.6 | W | 8.6~17.2 | 0.00 | 0.00 | 0.99 | 0.00 | 0.00 |
| Back | 42.4~50.6 | W | 17.2~ 25.8 | 0.01 | 0.01 | 0.96 | 0.01 | 0.01 |
| Back | 42.4~50.6 | W | 25.8~34.4 | 0.00 | 0.00 | 0.99 | 0.00 | 0.00 |
| Back | 42.4~50.6 | W | > 34.4 | 0.01 | 0.01 | 0.96 | 0.01 | 0.01 |
| Back | 50.6~58.8 | A | > 34.4 | 0.01 | 0.01 | 0.96 | 0.01 | 0.01 |
| Back | 50.6~58.8 | W | <= 8 .6 | 0.01 | 0.49 | 0.01 | 0.49 | 0.01 |
| Back | 50.6~58.8 | W | 8.6~17.2 | 0.00 | 0.00 | 0.79 | 0.20 | 0.00 |
| Back | > 58.8 | AA | 8.6~17.2 | 0.01 | 0.01 | 0.01 | 0.96 | 0.01 |
| Back | > 58.8 | W | <= 8.6 | 0.00 | 0.00 | 0.00 | 0.99 | 0.00 |
| Back | > 58.8 | W | 25.8~34.4 | 0.01 | 0.01 | 0.96 | 0.01 | 0.01 |
| ForeArm | <= 34.2 | H | > 34.4 | 0.01 | 0.01 | 0.96 | 0.01 | 0.01 |
| ForeArm | <= 34.2 | W | <= 8.6 | 0.00 | 0.99 | 0.00 | 0.00 | 0.00 |
| ForeArm | 34.2~42.4 | A | 17.2~25.8 | 0.01 | 0.01 | 0.96 | 0.01 | 0.01 |
| ForeArm | 34.2~42.4 | AA | > 34.4 | 0.01 | 0.01 | 0.96 | 0.01 | 0.01 |
| ForeArm | 34.2~42.4 | W | <= 8.6 | 0.33 | 0.33 | 0.00 | 0.33 | 0.01 |
| ForeArm | 34.2~42.4 | W | 25.8~34.4 | 0.01 | 0.01 | 0.96 | 0.01 | 0.01 |
| ForeArm | 42.4~50.6 | W | <= 8 .6 | 0.00 | 0.99 | 0.00 | 0.00 | 0.00 |
| ForeArm | 42.4~50.6 | W | 8.6~17.2 | 0.00 | 0.00 | 0.99 | 0.00 | 0.00 |
| ForeArm | 42.4~50.6 | W | 17.2~25.8 | 0.01 | 0.01 | 0.96 | 0.01 | 0.01 |
| ForeArm | 42.4~50.6 | W | 25.8~34.4 | 0.00 | 0.00 | 0.99 | 0.00 | 0.00 |
| ForeArm | 42.4~50.6 | W | > 34.4 | 0.01 | 0.01 | 0.96 | 0.01 | 0.01 |
| ForeArm | 50.6~58.8 | A | > 34.4 | 0.01 | 0.01 | 0.98 | 0.01 | 0.01 |
| ForeArm | 50.6~58.8 | W | < = 8.6 | 0.01 | 0.49 | 0.01 | 0.49 | 0.01 |
| ForeArm | 50.6~58.8 | W | 8.6~17.2 | 0.00 | 0.00 | 0.74 | 0.25 | 0.00 |
| ForeArm | 50.6~58.8 | W | 17.2~25.8 | 0.01 | 0.01 | 0.96 | 0.01 | 0.01 |
| ForeArm | > 58.8 | AA | 8.6 -17.2 | 0.01 | 0.01 | 0.01 | 0.96 | 0.01 |
| ForeArm | > 58.8 | W | < = 8.6 | 0.00 | 0.00 | 0.00 | 0.99 | 0.00 |
| ForeArm | > 58.8 | W | 17.2~25.8 | 0.01 | 0.01 | 0.96 | 0.01 | 0.01 |
| ForeArm | > 58.8 | W | 25.8~34.4 | 0.01 | 0.01 | 0.96 | 0.01 | 0.01 |
| Morphea | 42.4~50.6 | W | < = 8.6 | 0.00 | 0.00 | 0.00 | 0.00 | 0.99 |
| Morphea | 50.6~58.8 | W | <= 8.6 | 0.01 | 0.01 | 0.01 | 0.01 | 0.96 |
