# Supplementary figures and images for: Prediction of severity and subtype of fibrosing disease using model informed by inflammation and extracellular matrix gene index
Source: PLoS One. 2020 Oct 23;15(10):e0240986. doi: 10.1371/journal.pone.0240986 (PMC7584227; doi:10.1371/journal.pone.0240986)

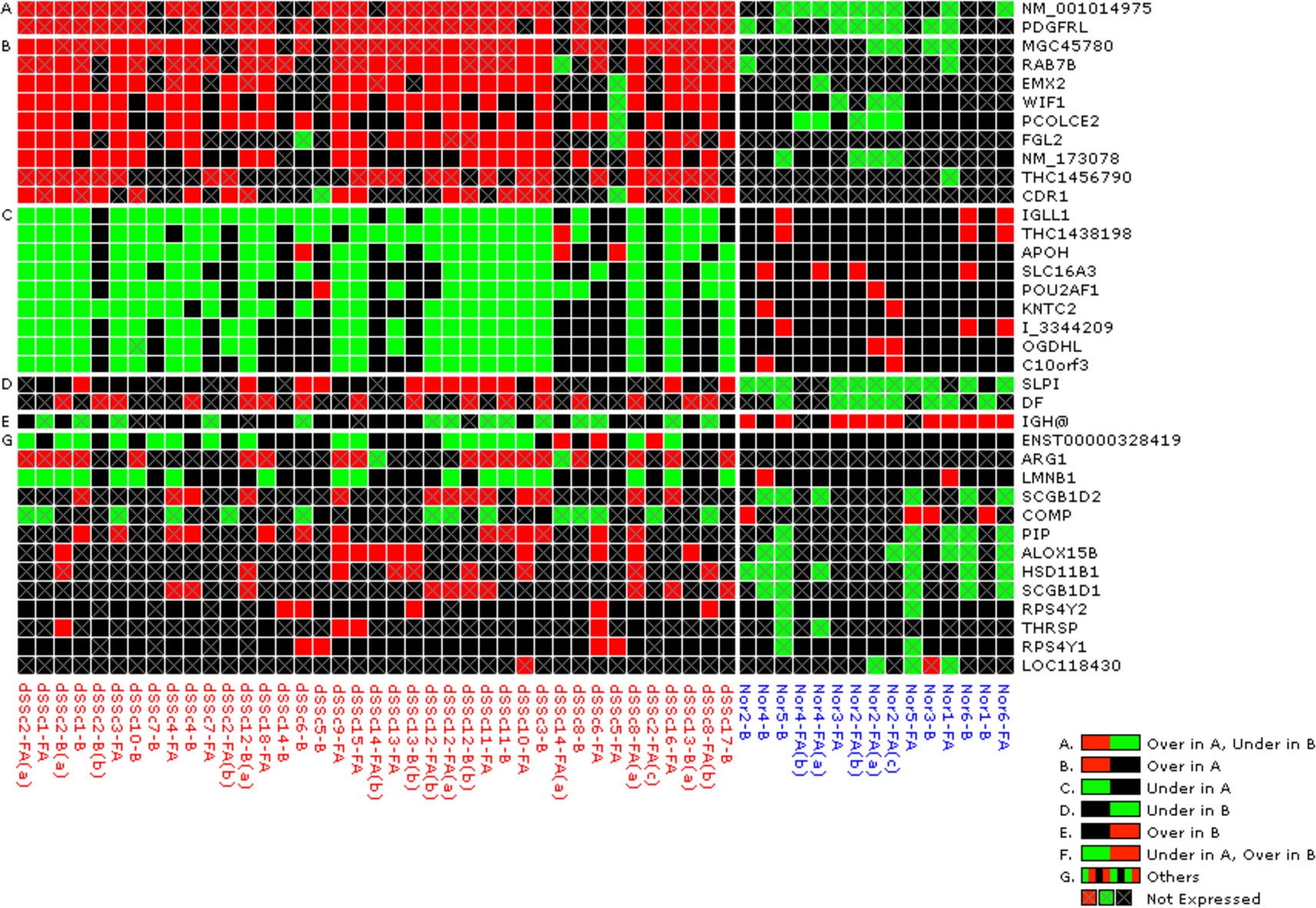

Supplement: S1 Fig — Color of boxes indicates directionality of expression differences with red indicating high expression and green indicating low expression. (TIF) [file pone.0240986.s003.tif]

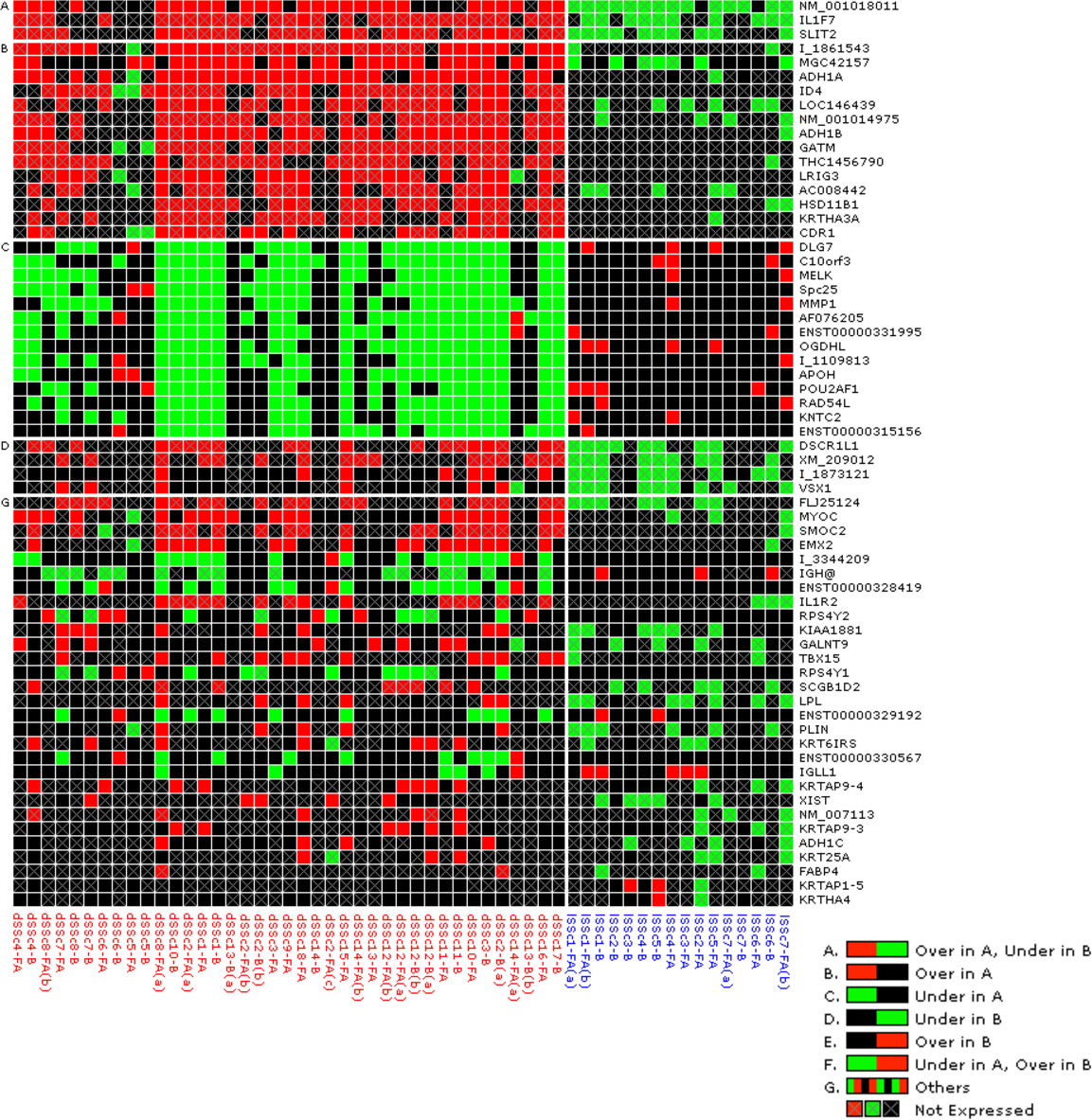

Supplement: S2 Fig — Color of boxes indicates directionality of expression differences with red indicating high expression and green indicating low expression. (TIF) [file pone.0240986.s004.tif]

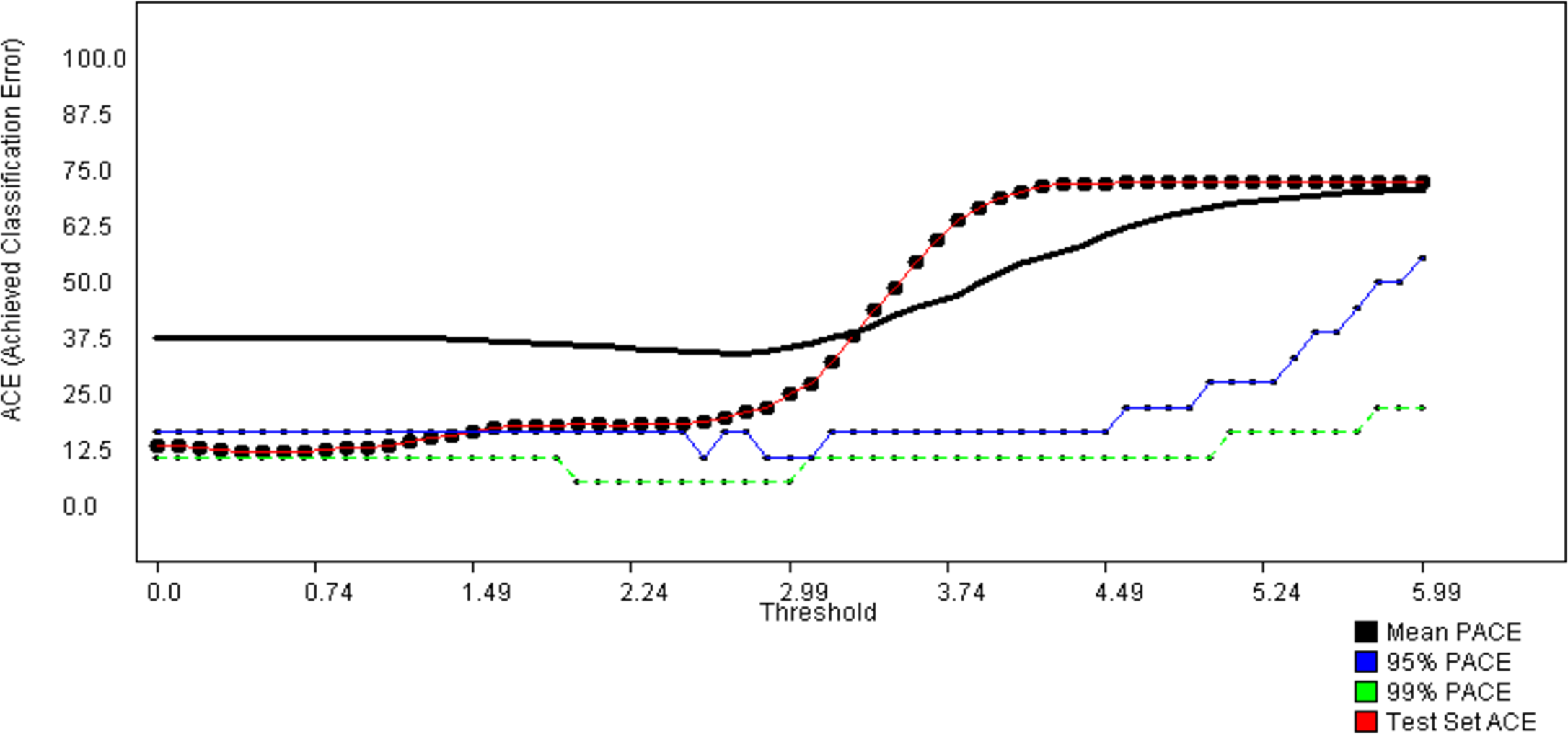

Supplement: S3 Fig — The model was significant at PACE 0.045 up to J5 1.4. (TIF) [file pone.0240986.s005.tif]

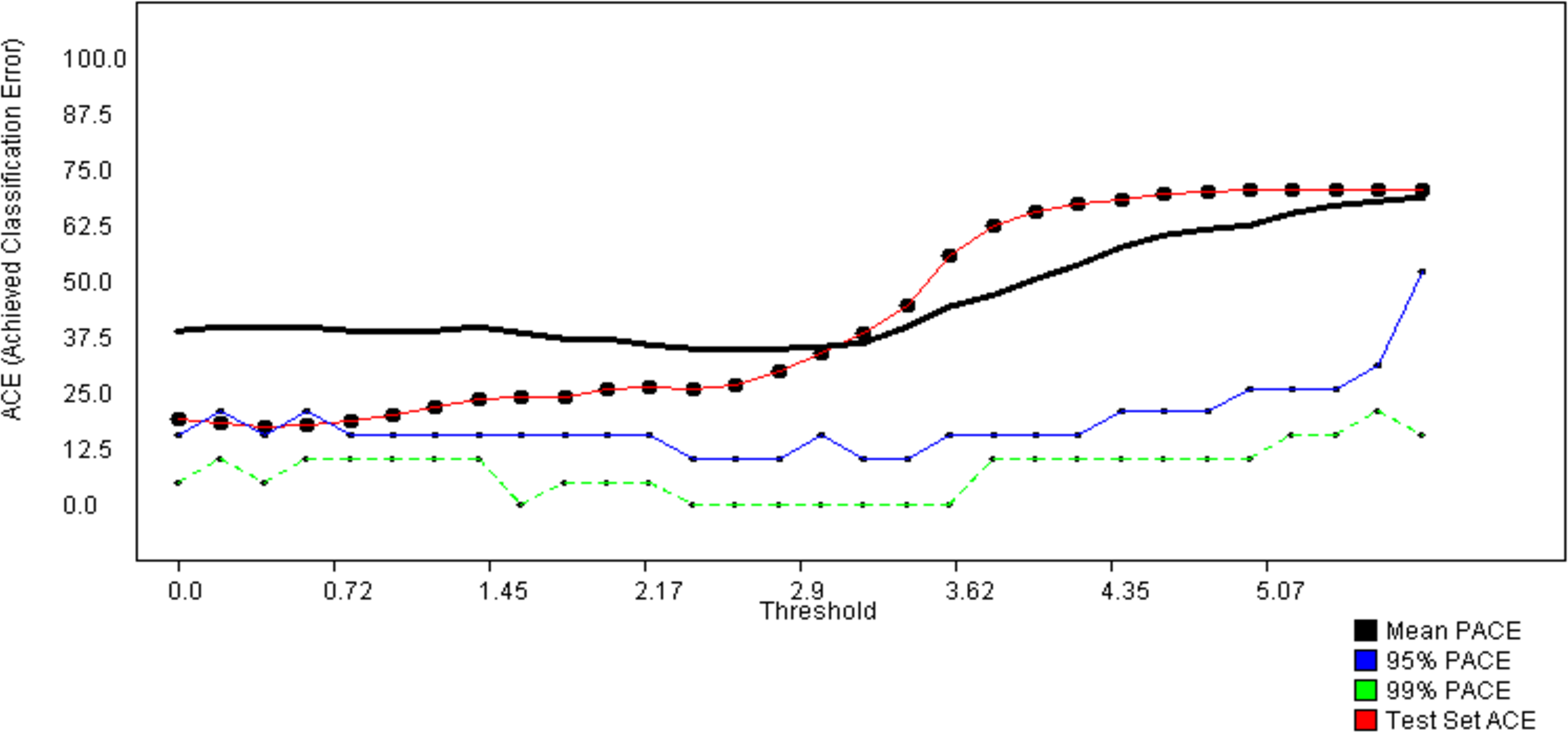

Supplement: S4 Fig — The model was significant at PACE 0.05 up to J5 1.1. (TIF) [file pone.0240986.s006.tif]
